# 

**Published:** 1885-09

**Authors:** 


### PAMPHLETS RECEIVED.

VOICE IN SINGERS. By Carl H. Von Kleim, A. M., M. D. Hann & Adair. Columbus, Ohio, 1885.

DRUGS AND MEDICINES OF NORTH AMERICA. By J. U. & C. G. Lloyd. Cincinnati, Ohio. Vol. I, No. 6.

